# Factors Associated With Candidemia in Hospitalised Patients in Mumbai, India: A Single Centre Retrospective Observational Study

**DOI:** 10.7759/cureus.117338

**Published:** 2026-10-01

**Authors:** Shashikala Shivaprakash, Neetu Biyani, Vasant Nagvekar, Pratiksha Chandorkar, Maninder S Setia

**Affiliations:** 1 Microbiology, Sir H. N. Reliance Foundation Hospital and Research Centre, Mumbai, IND; 2 Infectious Disease, Sir H. N. Reliance Foundation Hospital and Research Centre, Mumbai, IND; 3 Molecular Diagnostics, Sir H. N. Reliance Foundation Hospital and Research Centre, Mumbai, IND; 4 Epidemiology, Sir H. N. Reliance Foundation Hospital and Research Centre, Mumbai, IND

**Keywords:** antibiotics, candidemia, hospitalized patients, mortality, species

## Abstract

Introduction

Candidemia is an important bloodstream infection globally, and there is a variation in species across various regions of the world. Total parenteral nutrition, cancer, surgery, central venous catheterization, and exposure to antibiotics may be potential risk factors for candidemia. We conducted this study to assess the factors associated with Candidemia in hospitalized patients, study the mortality rates and factors associated with mortality, and describe the species in those with candidemia.

Methods

This was a single-center, retrospective, observational study of 139 patients who had candidal growth (cases) on blood culture and 259 patients who did not have any candidal growth (controls) on blood culture (2016-2023) in Mumbai, India. All the blood cultures were processed using the BACT/ALERT^®^ system (bioMérieux, France), and Candida isolates were identified using the VITEK^®^2 compact system and Matrix-Assisted Laser Desorption/ionization Time-Of-Flight mass spectrometry (MALDI-TOF MS, bioMérieux, France). We used logistic regression models for multivariate analysis, estimated the mortality rates in the study population, and used the Cox proportional hazards models to estimate the hazard ratios for mortality in the study population.

Results

The mean (SD) was 52.3 (24.1) years in cases and 56.1 (20.6) years in controls; the difference between the two groups was not statistically significant (p=0.11). In the multivariate analysis, factors associated with candidemia were: patients on inotropes (adjusted Odds Ratio [aOR]: 2.51, 95% confidence intervals [CI]: 1.35, 4.64; p<0.01), those in isolation (aOR: 2.11, 95% CI: 1.23, 3.62; p<0.01), and those who were on total parenteral nutrition for more than five days (aOR: 3.70, 95% CI: 1.80, 7.62; p<0.001). Candidemia was significantly lower in those who were on cephalosporins (aOR: 0.18, 95% CI: 0.06, 0.52; p<0.01). The mortality rate in the case group was 1.51 per 100 person-days (PD) (95% CI: 1.19, 1.91); it was higher compared with the control group (1.18 per 100 PD, 95% CI: 0.92, 1.52). After adjusting for demographic and clinical covariates, the hazard for mortality was higher in the candidemia group compared with controls (Hazard Ratio [HR]: 1.43, 95% CI: 0.93, 2.11), though this HR was not statistically significant (p=0.075). Species distribution revealed that *Candida tropicalis* (31 isolates) and *Candida auris* (29 isolates) were the most common, followed by *Candida albicans* (25 isolates).

Conclusions

In our study, non-albicans species were the most common type of organism, with *Candida tropicalis* and *Candida auris* being the most common species. The odds of candidemia were significantly higher in patients who were on total parenteral nutrition, those who were on inotropes, and those who were in isolation. In our total population, even though the hazard for mortality was higher in those with candidemia, it was not statistically significant. This was a single-centre retrospective observational study, and temporal changes in diagnostic methods, infection control practices and antifungal prescribing patterns over this period may have influenced the findings. There is a need to identify high-risk patients in hospital settings, have strict infection control practices and ensure appropriate and timely removal of invasive devices. Furthermore, there is a need for epidemiological surveillance of Candida species and antifungal resistance patterns in healthcare settings in India.

## Introduction

Candidemia is an important bloodstream infection globally; the incidence of bloodstream Candida infections has been reported to vary from 0.47 to 0.715 per 1000 patients [1-3]. A review found that though the total number of deaths due to candidemia had increased from 134,698 cases in 1990 to 174,716 in 2021, the age-standardized mortality rates (ASMR) had decreased during the same time period [4]. Furthermore, the ASMR showed substantial regional variation - Sub-Saharan Africa had high ASMR, and Europe, Australia, and the United States and Canada had lower ASMR; two countries, India and China, accounted for nearly 30% of deaths due to candidemia in 2021 [4]. In addition to epidemiological variability, there is also a variation in the species pattern across the globe. For instance, Indian studies have reported non-albicans species (such as *Candida parapsilosis* or *Candida tropicalis*) to be common [5, 6]. However, authors from European and North American studies have reported Candida albicans to be the most common species [1, 7, 8], even though others have found non-albicans species to be common in adults and pediatric patients [2]. It has also been reported that the proportion of Candida albicans is reducing globally, whereas those of *Candida parapsilosis* and *Candida tropicalis* are increasing [9]. Due to commercially available identification methods, Candida species are being tested and reported globally [10].

The underlying health condition of the patient and critical illness may be important risk factors for candidemia in adults [11]. Total parenteral nutrition appears to be an important risk factor for Candida bloodstream infection in patients admitted to the intensive care units (ICU) as well as non-ICU settings [12, 13]. Other risk factors include cancer, surgery, central venous catheter, and prior septic shock [12-14]. Studies have also compared the risk factors for albicans and non-albicans species separately [15, 16]. Though some risk factors may be similar for both, a study has reported that total parenteral nutrition was associated with a lower risk for non-albicans infection compared with Candida albicans [17]. Furthermore, Candida colonization of the urinary tract or respiratory tract may also be a risk factor for bloodstream infections [12]. Broad-spectrum antibiotic use is also an important risk factor for candidemia, including fluconazole-resistant infection [12, 18, 19]. Various studies have reported that exposure to antibiotics such as lipopeptides, glycopeptides, oxazolidinones, carbapenems, clindamycin, and colistin may be associated with a higher risk of infection [19, 20]. Some authors, however, have not found any independent association between antibiotic use and candidemia [21].

Candidemia is also associated with longer duration of hospital stay, including prolonged stay in the intensive care unit [22, 23]. Indeed, a study reported that patients with candidemia had an additional stay of 5.6 days in the intensive care unit [24]. The cost of treating the infection, as well as prolonged stay in the hospital, may increase the overall cost of management of these infections [25-29]. Thus, prevention and appropriate management of candidemia are required for reduction in morbidity, mortality, and overall cost of hospital care. As discussed earlier, India has a high burden of candidemia [4], and it is important to understand the factors associated with candidemia in hospital settings. Multiple factors may be present in hospitalized patients (such as total parenteral nutrition, catheterization or use of antibiotics). Furthermore, mortality in hospitalized patients may be associated with candidemia or other factors present in these patients. Thus, adjusting for these potential risk factors and confounders will be useful to understand the independent association of risk factors and mortality. With this background, we conducted this study: 1) to assess the factors associated with candidemia in hospitalized patients; 2) to study the mortality rates and factors associated with mortality in these patients; and 3) to describe the species and antifungal sensitivity in those with candidemia.

## Materials and methods

This was a single-center, retrospective, observational study of 139 patients who had growth of Candida species on blood culture and 259 patients who did not have any growth of Candida species on blood culture (2016-2023).

Study site and population

The study was conducted at a tertiary care centre in Mumbai, India. The hospital has all specialty services, including medical and surgical intensive care units. Only patients admitted to the hospital were eligible for inclusion in this study. If the blood culture was collected from the outpatient department, then they were not eligible for inclusion in the present study. A ‘case’ was a positive culture growth of Candida on blood culture. We included two blood cultures negative for Candida and were sent +/- 3 days of the positive Candida culture from the same location as the case (example, ward or intensive care unit) for the control group. The blood culture which was closest (day-wise) from the ‘case’ was selected for 'controls'. An individual could be selected only once as a control. An episode of candidemia was defined as the isolation of Candida species from one or more blood cultures along with features. The onset of candidemia was defined as the day when the first positive blood culture for Candida species was drawn from the patient. If the culture showed more than one growth, then these were also recorded in the data sheet.

All the blood cultures were processed using the BACT/ALERT® system (bioMérieux, Lyon, France), and Candida isolates were identified using the VITEK®2 compact system and Matrix-Assisted Laser Desorption/ionization Time-Of-Flight mass spectrometry (MALDI-TOF MS, bioMérieux, Lyon, France). Antifungal susceptibility testing was performed using VITEK®2 fungal susceptibility cards, which evaluate susceptibility to five antifungal agents: amphotericin B, 5-flucytosine, fluconazole, itraconazole, and voriconazole. C. albicans ATCC 90028 and C. parapsilosis ATCC 22019 were used as quality control strains in a microbroth dilution (colorimetric microplate-based assay). Antifungal susceptibility testing was strictly conducted according to the manufacturer’s standardized protocols. The minimum inhibitory concentration (MIC) was determined using the Clinical and Laboratory Standards Institute (CLSI) M27-A3 microbroth dilution method [30]. When no breakpoints were available, the breakpoints recommended by the antifungal susceptibility test kit/CDC (Centers for Disease Control and Prevention) were used for interpretation [31].

Study variables

Data were abstracted from clinical records of cases and controls. We included demographic data (age and sex), clinical diagnosis and treatment information. We also recorded date of admission, date of culture, and date of discharge or date of death. The total duration of hospital stay was calculated for each patient. We also collected information on antibiotics (number and type) the patients were receiving, co-morbidities and other risk factors such as hypertension, diabetes, ischemic heart disease, chronic kidney disease, malignancy, and organ transplant. Besides these, we also recorded data on invasive factors such as central venous catheterisation, mechanical ventilation, urinary tract catheterization, total parenteral nutrition, and intensive unit admission. We also collected information on surgeries (if any), immunosuppression, neutropenia, and whether the patient was kept in isolation in the hospital. The clinical details and risk factors were as of the day when the culture was collected and sent to the laboratory. Finally, we also included detailed information on the type of Candida species grown in the culture and antifungal sensitivity of these species.

Statistical methods

Data were entered in MS Excel (Microsoft Corp., Redmond, WA, USA) and converted to Stata Version 17 (StataCorp LLC, College Station, TX, USA). We estimated the means and standard deviations (SD) for normally distributed data and the median and interquartile range for data that were not normally distributed. We estimated the proportions for categorical variables. The means were compared using the t-test, and we used Mann-Whitney-Wilcoxon test for non-normal data. We used the chi-square test or Fisher’s exact test for low expected cell counts to compare the proportions across groups. We used logistic regression models for multivariate analysis to identify the factors associated with growth of Candida. The fit of the logistic models was assessed using the Akaike Information Criteria (AIC) and Bayesian Information Criteria (BIC) [32-34]. We then estimated the mortality rates in the study population and used the Cox proportional hazards models to estimate the hazard ratios for mortality in the study population. The proportional-hazards assumption was tested, and since it was not violated (Global test p=0.993), we used the Cox proportional hazards models for analysis. The main outcome (death/alive) was as on the last observable time. The number of days from admission to the last observable time was used for calculation of time, and the primary exposure variable was ‘growth of candidemia (case) compared with no candidemia (control). Other variables such as demographic, medication use, clinical variables, and other risk factors were included in the model as potential confounders. The mortality rates across categories were compared using the Poisson regression models. A p-value of <0.05 was considered statistically significant.

The study was approved by the Institutional Ethics Committee of Sir HN Reliance Foundation Hospital and Research Centre on 04 May 2024 (Reference No: HNH/IEC/2024/OCS/PATH/138).

## Results

We present results from 398 patients (139 cases with growth of Candida species and 259 controls who did not have any candidal growth on culture). Of these 139 cases, 10 patients had growth of two different types of Candida species, whereas all others had only one type of growth. The mean (SD) age of cases was 52.3 (24.1) years, and it was 56.1 (20.6) years in controls; the difference between the two groups was not statistically significant (p=0.11). There was no significant difference in the gender distribution among cases and controls.

Descriptive data and cross-tabulations

The majority of cases (38.1% [n=53]) and controls (44.8% [n=116]) were in the age group of 40 to < 60 years. In our study, the median number of antibiotics in cases was 2 (IQR: 1, 3), and it was 1 (IQR: 1, 2) in controls; the difference was statistically significant (p<0.001). More specifically, cases had a significantly higher proportion of the use of carbapenems, colistin/polymyxin, cephalosporins, monobactam, and lipopeptides compared with controls (Table 1). We also found that a significantly higher proportion of cases were on inotropes (64.8% [n=90] vs 39.8% [n=103]; p<0.001) and dialysis (39.6% [n=55] vs 21.6% [n=56]; p<0.001) compared with controls. The other risk factors that had a significantly higher proportion in cases were: surgery, isolation unit in the hospital, central venous catheterization for >7 days, urinary catheterization >2 days, total parenteral nutrition for >5 days, mechanical ventilation for >2 days and stay in the intensive care unit for >3 days. Complete details of proportions in cases and controls are presented in Table 1.

**Table 1:** Table showing the factors associated with Candida growth in 398 patients. The p-values were based on the chi-square test or Fisher's exact test for low expected cell counts for proportions. For non-normal data, we used the Mann-Whitney-Wilcoxon test. The test statistic (chi-square (χ2), t-test or z value has been presented. The data are presented as Mean and Standard Deviation (SD) or Median and Interquartile Range (IQR) for linear variables. The data are presented as N and % for categorical variables. The statistical significance is at p<0.05. SD: Standard Deviation; IQR: Interquartile range.

	Total	Candida growth	No growth	Test statistic	P value
	N (%)	n (%)	n (%)		
All	398 (100.0)	139 (34.9)	259 (65.1)		
Age (years)					
Mean (SD)	54.7 (21.9)	52.3 (24.1)	56.1 (20.6)	t=1.616	0.11
Age groups					
0 to 18	31 (7.8)	16 (11.5)	15 (5.8)	χ^2 ^= 5.529	0.24
18.1 to <40	52 (13.1)	17 (12.2)	35 (13.5)		
40 to <60	115 (28.9)	40 (28.8)	75 (28.9)		
60 to < 80	169 (42.5)	53 (38.1)	116 (44.8)		
>=80	31 (7.8)	13 (9.4)	18 (6.9)		
Gender					
Male	260 (65.3)	91 (35.0)	169 (65.0)	χ^2 ^= 0.002	0.97
Female	138 (34.7)	48 (34.8)	90 (65.2)		
Risk Factors					
Number of Antibiotics					
Median (IQR)	2 (1, 2)	2 (1, 3)	1 (1, 2)	z= 3.490	<0.001
Type of antibiotic (Yes)					
β lactam/lactamase inhibitors	165 (41.5)	63 (45.3)	102 (39.4)	χ^2 ^= 1.316	0.25
Carbapenem	131 (32.9)	51 (41.7)	73 (28.2)	χ^2^= 7.512	0.006
Colistin/Polymyxin	56 (14.1)	31 (22.3)	25 (9.7)	χ^2 ^= 11.971	0.001
Cephalosporins	46 (11.6)	7 (5.0)	39 (15.1)	χ^2 ^= 8.888	0.003
Glycopeptides	54 (13.6)	23 (16.6)	31 (11.9)	χ^2^ = 1.616	0.20
Monobactam	17 (4.3)	10 (7.2)	7 (2.7)	χ^2 ^= 4.463	0.035
Lipopeptide	11 (2.8)	7 (5.0)	4 (1.5)	χ^2^= 4.103	0.043
Others	82 (20.6)	37 (26.6)	45 (17.4)	χ^2 ^= 4.725	0.03
Other risk factors (Yes)					
Inotropes	193 (48.5)	90 (64.8)	103 (39.8)	χ^2 ^= 22.598	<0.001
Chronic Kidney Disease	93 (23.4)	42 (30.2)	51 (19.7)	χ^2 ^= 5.595	0.018
Diabetes Mellitus	184 (46.2)	60 (43.2)	124 (47.9)	χ^2^ = 0.808	0.37
Dialysis	111 (27.9)	55 (39.6)	56 (21.6)	χ^2 ^= 14.486	<0.001
Hypertension	226 (56.8)	78 (56.1)	148 (57.1)	χ^2 ^= 0.039	0.84
Ischemic Heart Disease	107 (26.9)	39 (28.1)	68 (26.3)	χ^2 ^= 0.149	0.70
Malignancy	86 (21.6)	32 (23.0)	54 (20.9)	χ^2 ^= 0.252	0.62
Metastases	46 (11.6)	19 (13.7)	27 (10.4)	χ^2 ^= 0.931	0.33
Gastrointestinal Carcinoma	39 (9.8)	18 (12.9)	21 (8.1)	χ^2^ = 2.399	0.12
Chemotherapy	66 (16.6)	25 (17.9)	41 (15.8)	χ^2 ^= 0.304	0.58
Transplant	46 (11.6)	19 (13.7)	27 (10.4)	χ^2 ^= 0.931	0.33
Hospitalisation related					
Surgery done	199 (50.3)	83 (60.1)	116 (44.9)	χ^2 ^= 8.291	0.004
Immunosuppression	220 (55.4)	83 (60.1)	137 (52.9)	χ^2 ^= 1.914	0.17
Neutropenia	18 (4.5)	7 (5.0)	11 (4.3)	χ^2 ^= 0.130	0.72
Isolation	126 (31.7)	65 (46.8)	61 (23.6)	χ^2 ^= 22.523	<0.001
Central Venous Catheter > 7 days	222 (55.8)	99 (71.2)	123 (47.5)	χ^2 ^= 20.655	<0.001
Urinary Catheter > 2 days	277 (69.6)	111 (79.9)	166 (64.1)	χ^2 ^= 10.622	0.001
Total parenteral nutrition >5 days	58 (14.6)	39 (28.1)	19 (7.4)	χ^2 ^= 31.007	<0.001
Mechanical Ventilation > 2 days	161 (40.5)	71 (51.1)	90 (34.8)	χ^2 ^= 10.014	0.002
Intensive Care Unit stay > 3 days	262 (65.8)	106 (76.3)	156 (60.2)	χ^2 ^= 10.329	0.001
Death					
Yes	130 (32.7)	70 (50.4)	60 (23.2)	χ^2 ^= 30.413	<0.001

Logistic regression models

In the multivariate analysis, after adjusting for potential confounders the following factors were significantly associated with candidemia: patients on inotropes (adjusted Odds Ratio [aOR]: 2.51, 95% confidence intervals [CI]: 1.35, 4.64; p<0.01), those in isolation (aOR: 2.11, 95% CI: 1.23, 3.62; p<0.01), and those who were on total parenteral nutrition for more than five days (aOR: 3.70, 95% CI: 1.80, 7.62; p<0.001). Candidemia was significantly lower in those who were on cephalosporins (aOR: 0.18, 95% CI: 0.06, 0.52; p<0.01). It was also significantly lower in patients aged 18 to <80 years compared with those less than 18 years of age. However, there was no significant difference between >=80-year-old and those less than 18 years (Table 2). We have presented odds ratios and their 95% confidence intervals (both univariate [unadjusted] and multivariate [adjusted] analysis) for demographic and clinical factors associated with candidemia.

**Table 2:** Table showing estimates from Unadjusted and Adjusted Logistic Regression Models for factors associated with Candidemia in 398 patients. * p <0.05, ** p <0.01, *** p <0.001, † p=0.055. Akaike Information Criteria: 469.60, Bayesian Information Criteria: 596.92 The values are presented as Odds Ratios (OR) and their 95% confidence intervals (CIs). The statistical significance is at p<0.05.

Parameters	Unadjusted Models	Adjusted Models
	Odds ratios (95% Confidence Intervals)	Odds ratios (95% Confidence Intervals)
Age groups (years)		
0 to 18	Reference	Reference
18.1 to <40	0.46 (0.18, 1.13)	0.29 (0.09, 0.90)*
40 to <60	0.50 (0.22, 1.16)	0.26 (0.09, 0.74)*
60 to < 80	0.43 (0.20, 0.93)*	0.20 (0.07, 0.62)**
>=80	0.68 (0.25, 1.85)	0.43 (0.11, 1.68)
Gender		
Female	Reference	Reference
Male	1.01 (0.65, 1.56)	0.81 (0.48, 1.37)
Type of antibiotic (Yes)		
β lactam/lactamase inhibitors	1.27 (0.84, 1.94)	1.11 (0.65, 1.91)
Carbapenem	1.82 (1.18, 2.81)**	1.29 (0.72, 2.32)
Colistin/Polymyxin	2.68 (1.51, 4.77)**	1.68 (0.81, 3.48)
Cephalosporins	0.29 (0.13, 0.69)**	0.18 (0.06, 0.52)**
Glycopeptides	1.46 (0.81, 2.61)	1.07 (0.51, 2.23)
Monobactam	2.79 (1.04, 7.50)*	2.20 (0.66, 7.38)
Lipopeptide	3.38 (0.97, 11.76)^†^	1.16 (0.25, 5.42)
Others	1.73 (1.05, 2.83)*	1.67 (0.91, 3.10)
Other risk factors (Yes)		
Inotropes	2.78 (1.81, 4.27)***	2.51 (1.35, 4.64)**
Chronic Kidney Disease	1.77 (1.10, 2.84)*	1.76 (0.82, 3.72)
Diabetes Mellitus	0.83 (0.55, 1.25)	0.84 (0.46, 1.52)
Dialysis	2.37 (1.51, 3.72)***	1.66 (0.86, 3.24)
Hypertension	0.96 (0.63, 1.45)	0.93 (0.49, 1.77)
Ischemic Heart Disease	1.09 (0.69, 1.74)	1.06 (0.54, 2.07)
Malignancy	1.14 (0.69, 1.86)	1.69 (0.65, 4.34)
Metastases	1.36 (0.73, 2.55)	--
Gastrointestinal Carcinoma	1.69 (0.87, 3.28)	--
Chemotherapy	1.17 (0.68, 2.01)	0.77 (0.25, 2.33)
Transplant	1.36 (0.73, 2.55)	0.77 (0.34, 1.78)
Hospitalisation related		
Surgery done	1.84 (1.21, 2.81)**	1.24 (0.71, 2.17)
Immunosuppression	1.34 (0.88, 2.04)	0.61 (0.35, 1.08)
Neutropenia	1.19 (0.45, 3.16)	1.63 (0.46, 5.74)
Isolation	2.85 (1.84, 4.43)***	2.11 (1.23, 3.62)**
Central Venous Catheter > 7 days	2.74 (1.76, 4.25)***	1.38 (0.70, 2.70)
Urinary Catheter > 2 days	2.22 (1.37, 3.61)**	1.32 (0.61, 2.86)
Total parenteral nutrition >5 days	4.91 (2.70, 8.90)***	3.70 (1.80, 7.62)***
Mechanical Ventilation > 2 days	1.96 (1.29, 2.98)**	0.70 (0.36, 1.35)
Intensive Care Unit stay > 3 days	2.12 (1.33, 3.37)**	0.75 (0.35, 1.59)

Mortality rates and Cox proportional hazards model

The mortality rate in the case group was 1.51 per 100 person-days (PD) (95% CI: 1.19, 1.91); it was higher compared with the control group (1.18 per 100 PD, 95% CI: 0.92, 1.52). The overall mortality rate was highest in patients >=80 years of age (3.11 per 100 PD, 95% CI: 2.03, 4.77). We found that, after adjusting for demographic and clinical covariates, the mortality hazard was higher in the candidemia group compared with controls (Hazard Ratio [HR]: 1.43, 95% CI: 0.93, 2.11), though this HR was not statistically significant (p=0.075). In the whole population, the hazard of mortality was significantly higher in those on inotropes (HR: 3.72, 95% CI: 2.09, 6.62, p<0.001). We have presented mortality rates, unadjusted hazard ratios, and adjusted hazard ratios along with their 95% confidence intervals in Table 3. We also estimated hazard ratios for those who had one growth and those who had two growths of Candida species. After adjusting for demographic and clinical covariates, the hazard was higher in those with one growth compared with no growth (HR: 1.45, 95% CI: 0.98, 2.16, p=0.063), even though this estimate was not statistically significant. However, there appeared to be no difference in those with two growths and those without any candidemia (HR: 1.01, 95% CI: 0.33, 3.13, p=0.98).

**Table 3:** Table showing the mortality rates, and estimates from unadjusted and adjusted Cox Proportional Hazards Models in 398 patients. * p <0.05, ** p <0.01, *** p <0.001, † p=0.058, ^a^ p=0.075, ^b^ p=0.072, Proportion-hazards assumption Global test p=0.993 The unit for mortality rates and their 95% confidence interval is per 100 person-days. The other parameters are Hazard Ratios (HRs) and their 95% confidence intervals. The statistical significance is at p<0.05

	Mortality rate	Unadjusted Models	Adjusted Models
	Per 100 person-days	Hazard ratios (95% Confidence Intervals)	Hazard ratios (95% Confidence Intervals)
All	1.34 (1.13, 1.59)		
Growth			
Candidemia – No growth	1.18 (0.92, 1.52)	Reference	Reference
Candidemia – Growth	1.51 (1.19, 1.91)	1.34 (0.95, 1.91)	1.43 (0.93, 2.11)^a^
Age groups			
0 to 18	0.97 (0.49, 1.94)	Reference	Reference
18.1 to <40	0.97 (0.53, 1.74)	0.90 (0.35, 2.28)	0.55 (0.19, 1.58)
40 to <60	0.85 (0.60, 1.22)	0.79 (0.36, 1.72)	0.47 (0.18, 1.23)
60 to < 80	1.68 (1.30, 2.18)	1.51 (0.72, 3.17)	1.08 (0.41, 2.82)
>=80	3.11 (2.03, 4.77)**	2.83 (1.25, 6.40)*	1.35 (0.46, 4.00)
Gender			
Female	1.32 (0.96, 1.80)	Reference	Reference
Male	1.35 (1.10, 1.65)	1.01 (0.69, 1.48)	1.27 (0.81, 1.98)
Type of antibiotic (Yes)			
β lactam/lactamase inhibitors	1.51 (1.18, 1.94)	1.21 (0.86, 1.71)	1.41 (0.89, 2.23)
Carbapenem	1.55 (1.19, 2.01)	1.31 (0.93, 1.86)	1.44 (0.91, 2.27)
Colistin/Polymyxin	1.47 (1.04, 2.07)	1.14 (0.76, 1.70)	1.30 (0.79, 2.13)
Cephalosporins	1.38 (0.82, 2.34)	0.99 (0.57, 1.73)	0.62 (0.31, 1.24)
Glycopeptides	1.36 (0.90, 2.05)	1.08 (0.68, 1.72)	1.16 (0.69, 1.96)
Monobactam	2.15 (1.22, 3.78)	1.50 (0.82, 2.72)	1.61 (0.75, 3.46)
Lipopeptide	1.71 (0.86, 3.43)	1.14 (0.56, 2.34)	0.81 (0.34, 1.88)
Others	1.56 (1.12, 2.16)	1.22 (0.82, 1.79)	1.38 (0.88, 2.14)
Other risk factors (Yes)			
Inotropes	1.74 (1.44, 2.11)***	2.53 (1.62, 3.94)***	3.72 (2.09, 6.62)***
Chronic Kidney Disease	1.72 (1.27, 2.33)^†^	1.35 (0.93, 1.96)	0.95 (0.53, 1.70)
Diabetes Mellitus	1.49 (1.18, 1.89)	1.26 (0.89, 1.78)	0.92 (0.59, 1.44)
Dialysis	1.60 (1.24, 2.08)	1.23 (0.86, 1.76)	1.25 (0.78, 2.02)
Hypertension	1.62 (1.31, 2.00)*	1.52 (1.05, 2.20)*	1.12 (0.66, 1.88)
Ischemic Heart Disease	1.59 (1.18, 2.12)	1.22 (0.85, 1.76)	0.78 (0.46, 1.31)
Malignancy	1.06 (0.73, 1.54)	0.74 (0.49, 1.13)	0.97 (0.43, 2.19)
Metastases	0.95 (0.57, 1.57)	0.72 (0.42, 1.24)	--
Gastrointestinal Carcinoma	1.68 (1.01, 2.79)	1.20 (0.70, 2.05)	--
Chemotherapy	1.07 (0.72, 1.60)	0.77 (0.49, 1.20)	0.59 (0.22, 1.55)
Transplant	1.17 (0.74, 1.83)	0.78 (0.48, 1.27)	1.43 (0.76, 2.71)
Hospitalisation related			
Surgery done	1.05 (0.83, 1.32)***	0.50 (0.35, 0.72)***	0.37 (0.23, 0.59)***
Immunosuppression	1.45 (1.18, 1.77)	1.26 (0.85, 1.86)	0.87 (0.53, 1.43)
Neutropenia	1.16 (0.55, 2.43)	0.77 (0.36, 1.65)	1.68 (0.64, 4.43)
Isolation	1.07 (0.80, 1.43)*	0.63 (0.44, 0.91)*	0.65 (0.41, 1.04)^b^
Central Venous Catheter > 7 days	1.13 (0.91, 1.39)**	0.40 (0.27, 0.59)***	0.23 (0.14, 0.40)***
Urinary Catheter > 2 days	1.40 (1.16, 1.68)	1.35 (0.78, 2.33)	1.17 (0.57, 2.41)
Total parenteral nutrition >5 days	1.09 (0.75, 1.59)	0.74 (0.48, 1.15)	1.24 (0.73, 2.12)
Mechanical Ventilation > 2 days	1.28 (1.03, 1.61)	0.86 (0.59, 1.25)	0.99 (0.60, 1.65)
Intensive Care Unit stay > 3 days	1.32 (1.10, 1.60)	0.83 (0.51, 1.35)	0.97 (0.51, 1.85)

Distribution of Candida species

A total of 149 Candida isolates representing 14 different Candida species were isolated during the study period, and non-albicans Candida species were the predominant isolates. Species distribution revealed that *Candida tropicalis* (31 isolates) and *Candida auris* (29 isolates) were the most common, followed by *Candida albicans* (25 isolates). Antifungal susceptibility showed high sensitivity of *Candida tropicalis* and *Candida albicans* to fluconazole, voriconazole, amphotericin B, caspofungin, and micafungin, whereas *Candida parapsilosis* demonstrated lower sensitivity to fluconazole.

## Discussion

In this study, we isolated 14 different species of Candida from 149 isolates over the eight-year period. We found that candidemia was significantly higher in patients who were on inotropes, those who were in isolation, and those who were on total parenteral nutrition for more than five days. Candida growth was significantly lower in those who were on cephalosporins. Even though the hazard for mortality was higher in those with candidemia, it was not statistically significant. Non-albicans Candida species were the predominant isolates during this eight-year period. *Candida tropicalis* and *Candida albicans* showed high antifungal susceptibility to fluconazole, voriconazole, amphotericin B, caspofungin, and micafungin.

Candidemia has been recognized as an important infection in hospital settings and may be associated with increased morbidity and mortality, particularly in intensive care settings [35-37]. In our study, factors significantly associated with candidemia were: patients on total parenteral nutrition, patients in isolation, and those on inotropes. Previous studies have also identified total parenteral nutrition as an independent risk factor for candidemia in ICU and non-ICU patients [13, 38-41]. Luzzati et al. reported a strong association of candidemia with total parenteral nutrition, with an odds ratio of 20.09 [42]. Other authors have reported total parenteral nutrition (TPN) to be a risk factor for candidemia even in neonates [18, 43]. It has been suggested that growth of Candida and biofilm formation may be due to the presence of fatty acids in the parenteral nutrition [18, 44]. Even though the use of inotropes was identified as an independent risk factor in our study, Lee et al. found that the risk of invasive candidiasis was significantly lower in infants who were on inotropes, and the adjusted odds ratio in their study was 0.4 (95% CI: 0.2 to 0.9) [45]. Nonetheless, the use of inotropes or total parenteral nutrition may be indicative of a more serious underlying condition, and it has been recommended that specific guidelines may be framed for the use of antifungals in patients who are on parenteral nutrition [38, 46]. Though other studies have also reported risk factors such as immunosuppression, intravascular lines, surgical procedures, urethral catheters, mechanical ventilation [13, 18, 38, 40, 47], these were not statistically significant in our study.

The use of broad-spectrum antibiotics has been reported to be an important risk factor for candidemia in patients [42, 47]. For instance, Munshi et al. reported that >14 days use of antibiotics was a risk factor for candidemia [40]. Additionally, other authors have shown that beta-lactam treatment, anti-MRSA therapy, and vancomycin were associated with candidemia [48, 49]. Dougherty et al. reported a higher hazard of candidemia with carbapenems compared with beta-lactams, whereas Ben-Ami et al. found that carbapenems, clindamycin, and colistin were associated with fluconazole-resistant Candida isolates [19, 50]. In our study, though the use of carbapenems, monobactams, and colistin/polymyxin was associated with higher candidemia growth in univariate models, these did not remain significant in the multivariate models. Though some other studies have shown a higher association of candidemia with cephalosporins [51, 52], we found a significantly lower association with candidemia. Dos Santos Rodrigues et al., in an in vivo model, found that cephalosporins showed antibiofilm activity and were able to reduce the fungal burden; thus, they have suggested the use of cephalexin and cefixime in infection control [53]. Though these in vitro findings may help us potentially explain the lower odds seen clinically in patients on cephalosporins, these findings have to be interpreted cautiously. There may be potential confounding by indication, severity of disease, or different prescription patterns.

Even though the mortality was higher in those with candidemia, the estimate was not statistically significant (though at p=0.075 it came close to the statistically significant cut-off of 0.05). The overall mortality in the candidemia group was 50.4%. This proportion is comparable to those reported by other authors. Some authors reported a higher proportion [54], whereas others have reported a lower mortality rate [23, 55, 56]. For example, the proportion of mortality reported by Sequeira et al. was 76.5% [54]. The proportion of mortality was 40.4% by Salmanton-Garcia et al., 26.6% by Hirano et al., and 29.6% by Li et al. [23, 55, 56]. In general, studies have shown that patients with candidemia who were on mechanical ventilation and total parenteral nutrition were at a greater risk for mortality [23, 56, 57]. As discussed earlier, some factors associated with candidemia (such as total parenteral nutrition and use of inotropes) may reflect a more serious underlying health condition [38, 56]. In our analysis, we did adjust for these potential confounders, and the adjusted hazard ratios for mortality in those with candidemia were estimated. The predominance of non-albicans Candida (NAC) species observed in this study aligns with epidemiological trends reported in India and other Asian countries. In our study, the most common organisms were *Candida tropicalis* and *Candida auris*. Other Indian studies have found *Candida tropicalis* to be the most common isolate [6, 58, 59]. Studies from other Asian countries have provided a mixed picture - some studies have found *Candida albicans* to be the most common species, whereas other authors have reported non-albicans species to be common or showing an increasing trend [3, 57, 60-62]. However, many other studies and epidemiological data have reported *Candida albicans* to be the most common organism [8, 63-65]. This increasing predominance of non-albicans Candida species in some areas and resistance patterns may reflect a shifting epidemiological trend which requires active epidemiological vigilance and other treatment strategies.

This was a single-centre retrospective study, thus the findings have to be interpreted within these limitations. The epidemiological data may not be representative of other hospital settings or national-level data. We do not claim causality, and the adjusted odds ratios from logistic models should be interpreted as association. In addition, temporal changes in diagnostic methods, infection control practices, and antifungal prescribing patterns over this period may have also influenced the findings of the study. Though we included risk factors in the analysis, we did not have complete data about the timing of antifungal therapy and source control. We have analysed the risk factors for candidemia in hospitalized patients and estimated the hazard for mortality in all patients; however, we did not do a specific mortality analysis in patients with candidemia according to species in this manuscript. Other studies have analysed mortality in different Candida species [55, 60]. Growth of candidemia may be related to the overall health condition of the patient in the hospital, and this may also have been associated with higher mortality. We included the total hospital stay for mortality analysis; however, we did adjust for potential confounders in the multivariate models and found the adjusted mortality rate for patients with a growth of candidemia compared with those who did not. Furthermore, although we used multivariate logistic regression models and Cox proportional hazards models and adjusted for potential confounders, there may be residual confounding. Despite these limitations, this study has collected and analysed data over a period of eight years. We studied the factors associated with candidemia as well as mortality rates in hospitalized patients.

## Conclusions

Thus, in this retrospective observational study, non-albicans species were the most common type of organism - with *Candida tropicalis* and *Candida auris* being the most common species. In our analysis, the odds of candidemia were significantly higher in patients who were on total parenteral nutrition, those who were on inotropes, and those who were in isolation. It was significantly lower in patients who were on cephalosporins. However, the lower odds have to be interpreted cautiously as there may be potential confounding by indication, severity of disease, or different prescription patterns. In our total population, even though the hazard for mortality was higher in those with candidemia compared with those without Candida growth, it was not statistically significant. There is a need to identify high-risk patients in hospital settings, have strict infection control practices, and ensure appropriate and timely removal of invasive devices. Furthermore, early identification of candidemia, including speciation and initiation of appropriate anti-fungal therapies, may be necessary for optimal care of hospitalized patients. In addition, there is a need for epidemiological surveillance of Candida species and antifungal resistance patterns in healthcare settings in India.
